# Single-Cell Transcriptome Profiling Identifies Phagocytosis-Related Dual-Feature Cells in A Model of Acute Otitis Media in Rats

**DOI:** 10.3389/fimmu.2021.760954

**Published:** 2021-10-25

**Authors:** Yufang Rao, Dalin Zhong, Ke Qiu, Danni Cheng, Li Li, Yi Zhang, Minzi Mao, Wendu Pang, Daibo Li, Yao Song, Junhong Li, Yijun Dong, Wei Zhang, Haopeng Yu, Jianjun Ren, Yu Zhao

**Affiliations:** ^1^ Department of Otolaryngology-Head and Neck Surgery, and National Clinical Research Center for Geriatrics, West China Hospital, Sichuan University, Chengdu, China; ^2^ Institute of Clinical Pathology, West China Hospital, Sichuan University, Chengdu, China; ^3^ Research Core Facility of West China Hospital, Sichuan University, Chengdu, China; ^4^ West China Biomedical Big Data Center, West China Hospital, Sichuan University, Chengdu, China; ^5^ Medical Big Data Center, Sichuan University, Chengdu, China

**Keywords:** single-cell RNA sequencing, acute otitis media, middle ear mucosa, macrophage, intercellular crosstalk, dual-feature

## Abstract

**Background:**

The molecular mechanisms of acute otitis media (AOM) development, and the intercellular crosstalk within the multicellular ecosystem of AOM, are not clear.

**Methods:**

We established a model of AOM in rats (with normal rats as controls) and undertook single-cell RNA sequencing (scRNA-seq) for the middle-ear mucosa (MEM). Cell clustering and trajectory analyses were undertaken using Seurat and Monocle 2 packages in R software. Pathway analyses were done by gene set enrichment analysis (GSEA). Cell–cell interactions were inferred by CellChat. Cell scores were calculated to identify cells with dual-feature.

**Results:**

A total of 7023 cells from three samples of inflamed MEM and 5258 cells from three samples of healthy MEM underwent scRNA-seq, which identified 20 cell clusters belonging to eight major cell types. After exposure to lipopolysaccharide, the MEM underwent significant conversion of cell types characterized by rapid infiltration of macrophages and neutrophils. M2 macrophages seemed to play a key part in inflammatory intercellular crosstalk, which facilitated the maintenance and proliferation of macrophages, cell chemotaxis, and regulation of the proinflammatory activities of cytokines. Three rare cell clusters with phagocytosis-related dual-feature were also identified. They coexisted with professional phagocytes in the MEM, and displayed distinct immunoregulatory functions by maintaining a normal immune microenvironment or influencing inflammation progression.

**Conclusions:**

Macrophages might be the “master” initiators and regulators of the inflammatory response of the MEM to external stimuli. And their functions are fulfilled by a specific polarization status (M2) and sophisticated intercellular crosstalk *via* certain signaling pathways. Besides, the coexistence of professional phagocytes and non-professional phagocytes as well as their interplay in the MEM provides new clues for deciphering the underlying pathogenic mechanisms of AOM.

## Introduction

Acute otitis media (AOM) is a common disease among children. It poses a heavy health burden on clinical practice worldwide, especially in developing countries (1).

In healthy individuals, the middle ear (ME) cavity is lined with a modified respiratory epithelium with ciliated and secretory cells, along with underlying loose connective tissue and vasculature (2). Various cell types of the middle-ear mucosa (MEM), such as immune cells, stroma cells, vascular cells, and even melanocytes, have been identified through flow cytometry, histology, and electron microscopy (3–8). Recently, Ryan et al. reported previously underestimated cell diversity in the MEM of normal rats *via* single-cell RNA sequencing (scRNA-seq), which provided a new perspective for further understanding of the microenvironment of the normal MEM (9). However, the response of the MEM to acute inflammation at single-cell resolution has not been illustrated. This knowledge gap prevents further understanding of maintenance of homeostasis of the MEM microenvironment and related disease-specific pathophysiologic mechanisms.

Studies have shown (mainly through flow cytometry) that the number and types of inflammatory cells in the MEM of rats change significantly after exposure to proinflammatory stimuli (10–12). Besides, bulk RNA-seq of MEM tissue and ME effusion have revealed that those shifts in cell composition are accompanied by extensive modifications of gene expression and their related signaling pathways, and involve certain pathophysiologic processes (9, 13–16). However, the molecular features and alterations of certain cell clusters have yet to be investigated further using scRNA-seq.

It has been reported that macrophages are scarce in the healthy MEM but increase in number rapidly in ME effusions after acute bacterial infection, and play an important part in AOM pathogenesis (17). Regulated by the surrounding micro-environment, macrophages can acquire heterogenous characteristics comprising mainly proinflammatory classically activated macrophages (“M1” macrophages) and anti-inflammatory alternatively activated macrophages (“M2” macrophages) (18, 19) The dynamic balance between M1/M2 subpopulations shapes the fate of inflammation in various inflammatory diseases (18, 20). Thus, scRNA-seq is needed to further demonstrate the interplay between macrophages and other cell types in the inflamed MEM at single-cell resolution.

We undertook scRNA-seq to explore systematically the landscapes of MEM cells in healthy rats and rats suffering from AOM. In this way, we aimed to: (i) further clarify alterations in the MEM after exposure to proinflammatory stimuli and the intercellular “crosstalk” within a multicellular ecosystem; (ii) identify novel cell types with unique molecular features and functions.

## Materials and Methods

### Ethical Approval of the Study Protocol

All study procedures conformed to guidelines set by the US National Institutes of Health (Bethesda, MD, USA). The study protocol was approved (2019271A) by the Animal Ethics Committee of West China Hospital within Sichuan University (Chengdu, China).

### Regents and Antibodies

Details of all regents and antibodies utilized in this study are listed in **Table S1**.

### Animals

Sprague–Dawley rats were housed in a room at constant temperature (25°C) and relative humidity (55%) and exposed to a 12-h light–dark cycle in a specific pathogen-free facility at the Laboratory Animal Center in Chengdu Dashuo Biotechnology (Chengdu, China). All rats had free access to water and chow.

### Generation and Evaluation of the AOM Model

Ten (i.e., 20 ME bullae) male rats (*Rattus norvegicus*; 8–10 weeks) were divided randomly into the AOM group and control (untreated) group. Otoscopic examinations were undertaken before treatment for all rats to ensure that tympanic membranes were normal and ME effusion was absent. Subsequently, rats in the AOM group were anesthetized with 2% pentobarbital sodium (0.2 mL/100 g, i.p.). Then, lipopolysaccharide (LPS; Sigma–Aldrich, Saint Louis, MO, USA) was injected into the bilateral ME cavity through the tympanic membrane. About 72 h after LPS injection in the AOM group, three rats from each group were sacrificed. In order to reach the requirement of cell number constructing scRNA-seq libraries, the corresponding six ME bullae in each group were isolated carefully and mixed for preparation of single-cell suspensions, respectively. The remaining rats in each group were sacrificed and their ME bullae isolated for hematoxylin and eosin (H&E) staining and immunofluorescence staining.

### Preparation of Single-Cell Suspensions

The MEM was harvested, as described previously (9). The harvested MEM was dissociated with collagenase type I (1 mg/mL; catalog number, SCR103; Sigma–Aldrich) and Dispase^®^ II (2 mg/mL; neutral protease, grade II; 4942078001; Sigma–Aldrich) in Dulbecco’s phosphate-buffered saline containing Ca^2+^ and Mg^2+^ for 25 min in a 37°C shaking incubator at 100 rpm. The MEM was dissociated further in 1.5 mL of Accumax™ Cell Dissociation Solution (AM105; Innovative Cell Technologies, San Diego, CA, USA) for 8 min in a 37°C shaking incubator at 100 rpm to aid isolation of single cells. Dissociated cells were filtered with 70-μm cell strainers (BD Biosciences, Franklin Lakes, NJ, USA) to eliminate clumps, and incubated in 1 mL of Red Blood Cell Lysing Solution (BD Biosciences) to remove red blood cells. Finally, we utilized 35-μm cell strainers (BD Biosciences) to filter cells and collected them according to manufacturer recommendations. Cell viability >80% was required for subsequent construction of libraries.

### Library Preparation and Sequencing

Based on the HiSeq X™ platform (Illumina, San Diego, CA, USA), cells were labeled with sample tags from the Human Single-Cell Multiplexing Kit from BD Biosciences. Then, they were counted, multiplexed, and prepared for subsequent single-cell capture. The latter and complementary-DNA synthesis were undertaken by the Rhapsody Single-Cell Analysis System according to manufacturer (BD Biosciences) recommendations. Libraries were sequenced on multiple runs of NextSeq™ (Illumina).

### Quality Control and Preprocessing of Sequencing Data

Raw scRNA-seq data were processed according to a bioinformatics pipeline (BD Rhapsody™ WTA 1.9.1; https://bitbucket.org/CRSwDev/cwl/src/master/). This process included demultiplexing FASTQ reads, mapping to the rat genome (Rnor_6.0, STAR v2.5.2b), and generating gene/barcode matrices.

### Unsupervised Clustering of Cells and t-Distributed Stochastic Neighbor Embedding (tSNE) Visualization

R package Seurat v4.0 was used for downstream data analyses (21). Following a standard workflow, we filtered cells that had a unique feature count >4000 or <200 and cells with a mitochondrial count >20% in the inflamed-MEM group. We also filtered cells that had a unique feature counts >3000 or <200 and cells with a mitochondrial count >40% in the normal-MEM group. Then, the default parameters of the “NormalizeData” function of Seurat were used to normalize the feature-expression measurements for each cell by the total expression. Finally, 7024 cells of the case group and 5258 cells of the control group were introduced into a combined Seurat object *via* “FindIntegrationAnchors” and “IntegrateData” functions. Then, variable genes were carried forward into scaling and principal component (PC) analysis. Significant PCs (top 30) were used for t-SNE analysis and clustering through “RunTSNE” and “FindClusters” functions (resolution = 0.6). To identify cell types, we checked whether the well-studied marker genes were the top differentially expressed genes (DEGs), and annotated the most likely identity for each cell cluster. The remaining cell types were identified by manual retrieval from a database.

### Differential Expression and Analysis of Signaling Pathways

Differentially expressed genes (DEGs) among clusters were detected by the Seurat function “FindAllMarkers”. Selected functional DEGs of each cluster were visualized *via* stacked violin plots. Volcano plots were applied to show the genes with upregulated or downregulated expression. Heatmaps showing the expression distribution of marker genes of cell clusters were created by pheatmap 1.0.12 (R package). Genes with Benjamini–Hochberg-adjusted P < 0.05 and absolute log_2_fold change between two groups >2 were used for analysis of fuctional enrichment using the Gene Ontology (GO) database (clusterProfiler 3.16.1). In addition, ranked gene set enrichment analysis (GSEA) was undertaken: genes were ranked based on their phenotypes, and the GSEA algorithm proposed by Subramanian and coworkers (Subramanian et al., 2005) was used to calculate the enrichment score of each gene.

### Trajectory Analyses

We used Monocle 2 v2.5.4 (R package) (22) to order single cells in “pseudotime”, and placed them along an inferred trajectory. After passing quality control, genes were placed into the Reversed Graph Embedding algorithm of Monocle to shape the trajectory. Then, Monocle applied a dimensionality reduction to the data and ordered the cells in pseudotime.

### Ligand–Receptor Expression and Cell Interactions

Cell-to-cell communication (CellChat 0.0.2; R package) was ascertained by evaluating expression of pairs of ligands and receptors within cell populations (23, 24). We examined the interaction between different cell types, and gene expression of 0.2 was set as the valid cutoff point.

### Cell Score (CS) Calculation

The CS denotes the mean expression of specific gene functions (e.g., scores for the cell cycle, M1 macrophages, M2 macrophages, epithelia) in single cells. The CS was calculated, as described previously (25, 26). Briefly, a control gene set (Gc) was defined and genes were partitioned into 25 “bins” according to their mean expression across all cells. Then, for each gene from the target gene set (Gt), 100 genes were selected randomly from the bin to which this gene belonged to constitute the Gc. Therefore, the CS was calculated as mean (Gt) – mean (Gc). Relative expression was used to calculate the CS.

### Multiplex Immunofluorescence

Two representative bullae from AOM group and control group were carefully isolated, and then fixed and embedded them with formalin and paraffin, respectively. The formalin fixed paraffin embedded bullae were sectioned and processed by using Opal Polaris™ 7-color Manual IHC Kit (Akoya Biosciences) as the manufacturer’s recommendation (27). Specifically, the opal panel included DAPI (Abcam), anti-CD68 (Abcam), anti-EPCAM (Abcam), anti-MKI67 (Abcam), anti-COL1A1 (Abcam) and anti-SFRP4 (Abcam).

## Results

### Single-Cell Transcriptome Profiling of the MEM in Rats

**Figure 1:**
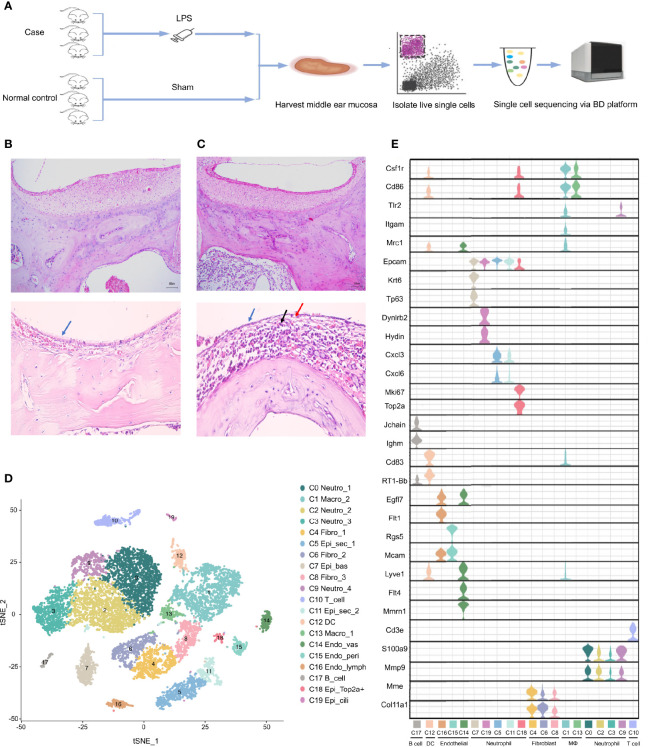
Single cell transcriptome profiling of rat middle ear mucosa (MEM). **(A)** Workflow showing the process of model construction, sample collection and scRNA-seq using BD Rhapsody platform. **(B)** H&E staining of normal MEM. Normal epithelium is indicated by the bule arrow. **(C)** H&E staining of inflamed MEM. Damaged epithelium is indicated by the blue and red arrow. Inflammatory infiltration is indicated by the black arrow. **(D)** t-SNE plot of all single cells from BD Rhapsody. **(E)** Violin plots showing the expression levels of different marker genes across 19 clusters.

To systematically investigate the cellular diversity of the inflamed MEM in rats as well as the sophisticated intercellular crosstalk within its multicellular ecosystem. A model of AOM in rats was established (**Figure 1A**), which was examined by histology using H&E staining (**Figures 1B, C**). Subsequently, we undertook scRNA-seq on 7023 cells from six samples of the inflamed MEM and 5258 cells from six healthy samples *via* BD WTA Rhapsody Analysis Pipeline 1.9.1 after quality control and removal of doublets (**Figure 1A** and **Table S2**).

Then, sequencing data from the 12 samples were integrated and analyzed using Seurat. Overall, the transcriptomes of eight major cell types were captured based on expression of known canonical gene markers (**Table S2**). Unsupervised clustering analysis categorized all cells further into 20 distinct clusters (**Figure 1D** and **Table S3**). Each of the 20 clusters possessed specifically expressed marker genes, which were consistent to their distinct cell identities, as described previously (**Figure 1E**, **Table S4** and **Supplementary Information 1**), including neutrophils (C0, C2, C3 and C9), macrophages (C1 and C13), fibroblasts (C4, C6 and C8), epithelial cells (C5, C7, C11, C18 and C19), T cells (C10), dendritic cells (C12), endothelial cells (C14, C15 and C16) and B cells (C17).

Overall, scRNA-seq of the MEM of rats revealed considerable cell diversity that was underestimated previously. Detailed marker gene lists were provided in **Table S2**.

### AOM Is Characterized by Rapid Infiltration of Neutrophils and Macrophages During Inflammatory Progression

**Figure 2:**
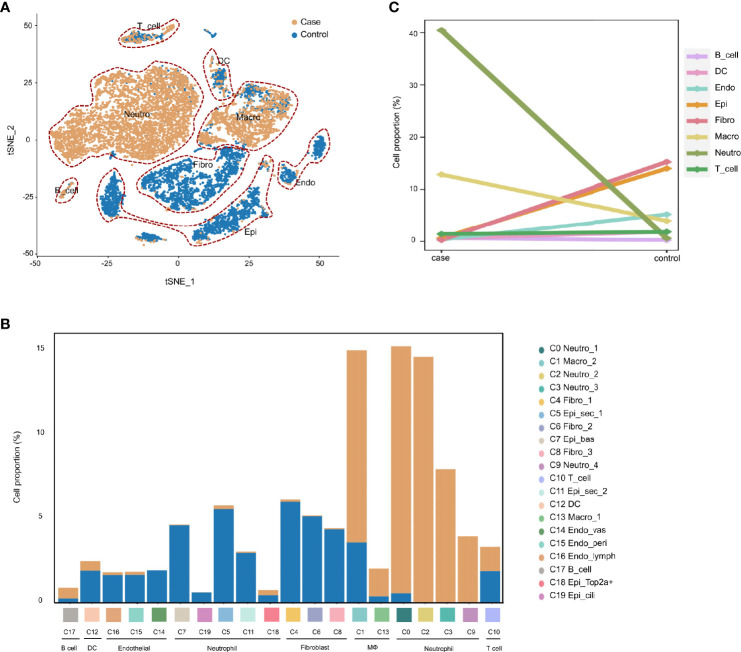
AOM is characterized by rapid infiltration of neutrophils and macrophages during inflammatory progression. **(A)** t-SNE plots comparing the distribution of single cells derived from inflamed and normal MEM **(B)** Comparison of cell proportions in each cluster between normal and inflamed MEM **(C)** Comparison of cell proportions in each major cell type between normal and inflamed MEM.

To demonstrate the conversion of cell types in the MEM of rats in response to proinflammatory stimuli. We compared the cell compositions between the normal MEM and inflamed MEM, of which obvious differences were identified. Epithelial cells (C0, C3, C12, C14, C15 and C17) and fibroblasts (C1, C2 and C4) were the major cell types of the normal MEM, which accounted for approximately two-thirds of all single cells. However, after exposure to LPS, rapid infiltration of innate immune cells, including neutrophils (C0, C1, C2 and C4) and macrophages (C3, C5 and C6) occurred, and overwhelmed the normally predominant cell types (**Figures 2A, B**). More specifically, compared with their counterparts from the normal MEM, an obvious increase in the number of neutrophils and macrophages as well as an obvious reduction in the number of fibroblasts and epithelial cells, were observed in inflamed MEM samples (**Figure 2C**).

Taken together, these data suggested that, after exposure to LPS, the MEM in rats underwent significant conversion of cell types, which was characterized by the rapid infiltration of macrophages and neutrophils.

### M2 Macrophages Are Key Regulators in Inflammation Progression

**Figure 3:**
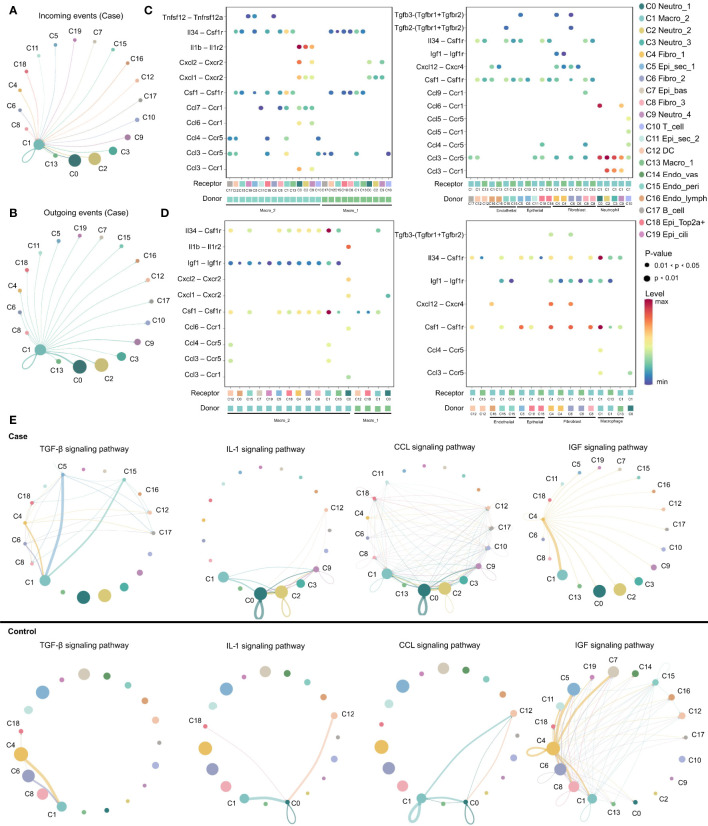
M2 macrophage play as a key regulator in inflammation progression. **(A, B)** Circos plots displaying putative ligand–receptor interactions between macro_2 and other cell clusters from inflamed MEM. Interactions are divided into incoming and outgoing events. The brand links pairs of interacting cell types, and corresponding number of events were labeled in the graph **(C, D)** Summary of selected ligand–receptor interactions between different cell clusters from inflamed MEM and normal MEM, respectively. P–values (permutation test) are represented by the size of each circle. The color gradient indicates the level of interaction. **(E)** The inferred signaling networks between different cell clusters. Circle sizes are proportional to the number of cells in each cell group and edge width represents the communication probability.

Expression of various ligand–receptor pairs was investigated to decipher the cell–cell interactions in the multicellular system of the normal MEM and inflamed MEM. In the inflamed MEM, the macro_2 cluster (C1), which specifically expressed *Mrc1* (canonical marker gene of M2 macrophages) and was polarized towards the M2 phenotype (**Table S4**), displayed vast communication with nearly all other cell clusters (especially those mediated by other macrophage clusters and neutrophils) and seemed to play a key part in inflammatory intercellular crosstalk (**Figures 3A, B**). We further investigated the specific ligand–receptor interactions among different cell clusters. In the inflamed MEM, we identified strong somatotrophic interactions among macrophages as well as between macrophages (especially macro_2) and other clusters, including colony-stimulating factor 1–colony stimulating factor 1 receptor (CSF1–CSF1R) and interleukin 34 (IL34)–CSF1R, which might mediate the maintenance and proliferation of macrophages after exposure to external stimuli (28). Besides, recruitment of neutrophils, natural killer cells, and T cells by macrophages (especially macro_2) *via* chemoattraction was observed (e.g., C-C motif chemokine ligand 3–C-C motif chemokine receptor 5 (CCL3–CCR5) and C-X-C motif chemokine ligand 1–C-X-C motif chemokine receptor 2 (CXCL1–CXCR2)). Specifically, interleukin 1 beta–interleukin 1 receptor 2 (IL1β–IL1R2) interactions between macrophages and neutrophils (which have been shown to be of vital importance in regulating the proinflammatory activities of IL-1 and contributing to the resolution of acute inflammation) (29) were detected (**Figure 3C**).

In the normal MEM, CSF1–CSF1R and IL34–CSF1R interactions were enhanced significantly between epithelial cells and macrophages (especially macro_2) as well as fibroblasts and macrophages (especially macro_2). These data suggested that epithelial cells and fibroblasts might be the main source of CSF1/IL34 under normal conditions, which has a crucial role in macrophage survival (28). Besides, inhibition of expression of proinflammatory phenotypes of potential non-professional phagocytes, including epithelial cells, fibroblasts, and endothelial cells, by macrophages (especially macro_2) *via* insulin like growth factor 1–insulin like growth factor 1 receptor (IGF1–IGF1R) interactions, was also observed, which might help to maintain the microenvironment of the normal MEM (**Figure 3D**) (30). Overall, these data suggested that M2 macrophages might play an important part in the MEM under normal conditions and inflammatory conditions.

Ligand–receptor interactions within certain signaling pathways were also predicted. Specific signaling pathways, including transforming growth factor beta (TGFβ), CCL, IGF and IL1, were identified. They displayed highly distinct cell-communication networks between the normal MEM and inflamed MEM, and might play an important part in the development and progression of AOM (**Figure 3E**) (20, 31–37). More detailed cell–cell interactions are presented in **Figure S2**.

Overall, M2 macrophages seemed to have a key role in inflammatory intercellular crosstalk, which facilitated the maintenance and proliferation of macrophages, cell chemotaxis, as well as regulation of the proinflammatory activities of cytokines.

### Phagocytosis-Related Dual-Feature Cells and Professional Phagocytes Coexist in the MEM

**Figure 4:**
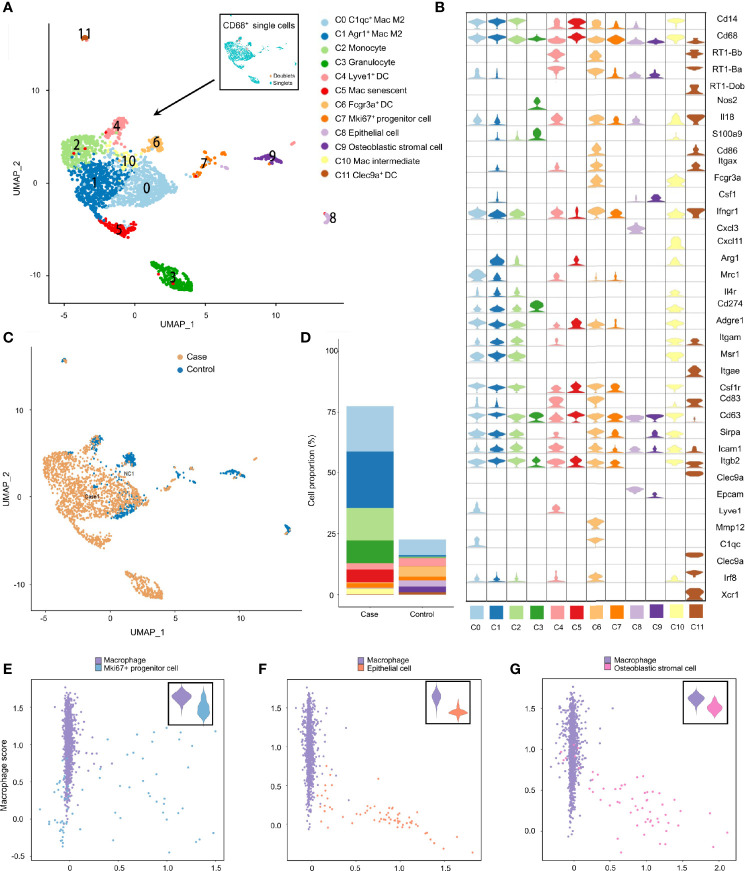
Phagocytosis-related dual-feature cells and professional phagocytes coexist in rat MEM. **(A)** UMAP plot of CD68+ single cells at the resolution of 0.6 as well as UMAP plot showing the distribution of singlets and doublets (indicated at the upper right corner) **(B)** Violin plots showing the expression levels of different marker genes across 12 clusters of CD68+ single cells **(C)** UMAP plots comparing the distribution of CD68+ single cells derived from inflamed and normal MEM **(D)** Comparison of cell proportions of each cluster between inflamed and normal MEM **(E)** Scatterplot showing the correlation between the Mki67+ progenitor cell scores and macrophage scores as well as comparison of macrophage scores of single cells between macrophages and Mki67+ progenitor cells **(F)** Scatterplot showing the correlation between the epithelial cell scores and macrophage scores as well as comparison of macrophage scores of single cells between macrophages and epithelial cells **(G)** Scatterplot showing the correlation between the fibroblast scores and macrophage scores as well as comparison of macrophage scores of single cells between macrophages and fibroblasts.

Studies have revealed the coexistence of professional phagocytes and non-professional phagocytes in multiple tissues, between which crosstalk disruption has been shown to be responsible for various diseases and might play an important part in the inflammatory response (38, 39). That hypothesis was bolstered in the present study. Specifically, besides professional phagocytes (neutrophils, macrophages and dendritic cells), other clusters from the inflamed MEM and normal MEM also displayed high expression of phagocytosis-related genes, such as *Cd14* and *Cd68* (**Tables S4** and **S6**), indicating that these cell clusters might have dual features. Therefore, to further confirm the existence of phagocytosis-related dual-feature cells in the MEM, we specifically extracted all *CD68* ^+^ single cells for subsequent analyses. Doublets were identified and excluded by using “DoubletFinder” 2.0.3 (R package). Clustering tree 0.4.3 revealed that a stable clustering strategy was achieved at a resolution of 0.6, which generated 12 clusters belonging to seven major cell types (**Figure S3**, **Figure 4A**, **Table S6–4** and **Supplementary Information 2**).

Apart from well-known professional phagocytes such as macrophages (C0, C1, C5 and C10), monocytes (C2), dendritic cells (C4, C6 and C11), and granulocytes (C3), three rare cell clusters (C7–C9) with dual features were also identified. Cluster C7 displayed high expression of cell cycle-related genes, including *Ccna2*, *Ccnb1* and *Cdk1*, as well as stem-like genes *Mki67* (marker gene of proliferation) and *Aspm* (involved in regulation of the mitotic spindle and coordination of mitotic processes), so cluster C7 comprised *Mki67* ^+^ progenitor cells (**Figure 4B** and **Tables S6–4**) (40). Cluster C8 specifically expressed *Epcam* (a gene associated with epithelial cells) and *Krt18* (cytokeratin gene), which strongly indicated that it was an epithelial cell (**Figure 4B** and **Table S6–4**). Cluster C9 showed high expression of genes related to the development and maintenance of bone, including *Bmp5* and *Cdh11*, as well as genes related to matrix homeostasis, including *Col5a2*, *Col12a1* and *Mmp2*, which is consistent with osteoblastic stromal cells (**Figure 4B** and **Tables S6–4**). Additionally, the majority of CD68^+^ cells derived from inflamed MEM and the cell compositions as well as cell proportions showed obvious differences between normal MEM and inflamed MEM (**Figures 4C, D**).

To further quantify the dual features of these non-professional phagocytes, we calculated the CS of target gene sets of selected cell clusters (*Mki67* ^+^ progenitor cells, epithelial cells, and osteoblastic stromal cells; expression of their selected marker genes is shown in **Figure S4A**). Some *Mki67* ^+^ progenitor cells had a high phagocytosis score and proliferation score (**Figure 4E**), which further demonstrated their phagocytic-proliferating dual-feature (**Tables S8-1**, **S8–2**). To a certain extent, some epithelial cells and osteoblastic stromal cells also showed trends of a dual feature related to phagocytosis (**Figures 4F, G** and **Tables S8–1**, **S8–3**, **S8–4**). The M1 score and M2 score were calculated to clarify the polarization status of the four subpopulations of macrophages. M2 was the main phenotype of these four clusters, whereas the gene signatures of M1 and M2 were not mutually exclusive. For example, many *C1qc* ^+^ macrophages also presented higher expression of M1 marker genes than the other three clusters (**Figures S4B, C**).

**Figure 5:**
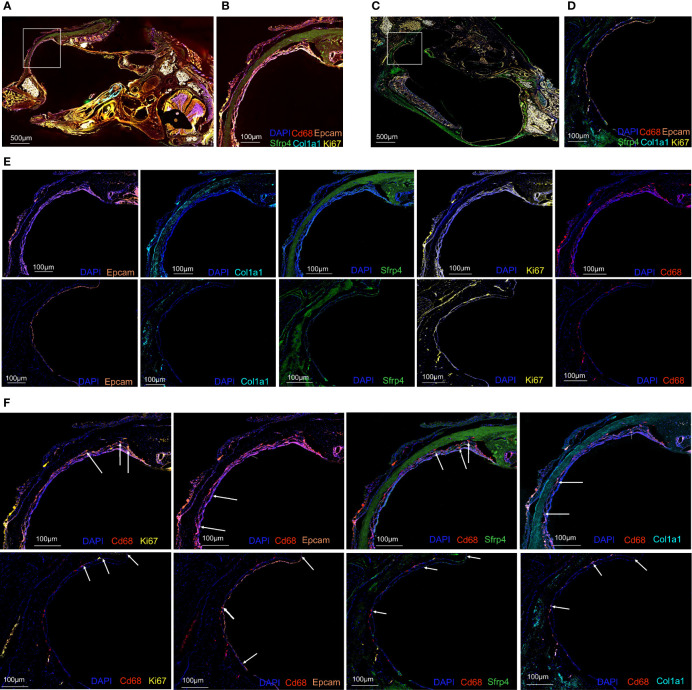
Multiplex immunofluorescence confirmed the existence of phagocytosis-related dual-feature cells in rat MEM **(A)** Landscape of rat inflamed MEM stained with antibodies against CD68, EPCAM, MKI67, COL1A1 and SFRP4 and counterstained with DAPI **(B)** Target region of rat inflamed MEM stained with antibodies against CD68, EPCAM, MKI67, COL1A1 and SFRP4 and counterstained with DAPI **(C)** Landscape of rat normal MEM stained with antibodies against CD68, EPCAM, MKI67, COL1A1 and SFRP4 and counterstained with DAPI **(D)** Target region of rat normal MEM stained with antibodies against CD68, EPCAM, MKI67, COL1A1 and SFRP4 and counterstained with DAPI **(E)** Target region of rat inflamed (upper images) and normal (lower images) MEM stained with antibodies against EPCAM, COL1A1, SFRP4, MKI67, CD68, and counterstained with DAPI. **(F)** Target region of rat inflamed (upper images) and normal (lower images) MEM stained with antibodies against CD68 and MKI67, CD68 and EPCAM, CD68 and SFRP4, CD68 and COL1A1, and counterstained with DAPI.

To further confirm the existence of phagocytosis-related dual-feature cells, multiplex immunofluorescence was conducted in both normal MEM (**Figures 5A, B, E**) and inflamed MEM (**Figures 5C–E**). And the results showed that certain cells in both normal MEM and inflamed MEM co-expressed phagocytosis marker *CD68* and proliferation marker *MKI67*, consistent with the definition of phagocytic-proliferating dual-feature cells (**Figure 5F**). Besides, the colocalization of *CD68* and epithelial marker *EPCAM* were observed in both normal and inflamed MEM, suggesting of cells with phagocytic-epithelial dual feature (**Figure 5F**). Similarly, the existence of phagocytic-fibroblastic dual-feature cells were also confirmed in both normal and inflamed MEM based on the co-expression of *CD68* and fibroblast marker *SFRP4* or *COL1A1* (**Figure 5F**).

Taken together, we identified three rare cell clusters with a phagocytosis-related dual feature. These cells are consistent with the definition of previously described non-professional phagocytes, and coexist with professional phagocytes in the MEM of rats.

### Phagocytosis-Related Dual-Feature Cells Are a Heterogeneous Group of Cells With Distinct Immunoregulatory Functions

**Figure 6:**
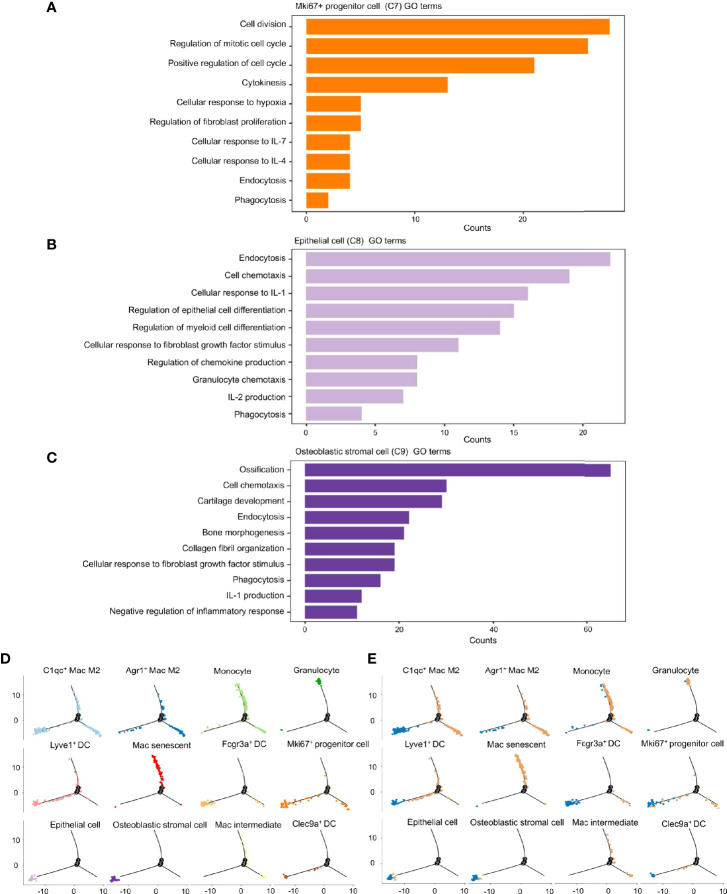
Phagocytosis-related dual-feature cells are a heterogeneous group of cells with distinct immune regulation functions. **(A)** Top canonical pathways enriched in the Mki67+ progenitor cell (cluster C7) **(B)** Top canonical pathways enriched in the epithelial cell (cluster C8) **(C)** Top canonical pathways enriched in the Osteoblastic stromal cell (cluster C9) **(D)** The developmental trajectory of cells in each cluster inferred by Monocle2 **(E)** The developmental trajectory of cells in each cluster inferred by Monocle2 comparing the distribution of CD68^+^ single cells derived from inflamed and normal MEM. The developmental trajectories showed that most of the Mki67+ progenitor cells, epithelial cells and osteoblastic stromal cells were located on the early branch, indicating their derivation from normal MEM as well as their failure to maintain self-integrity in the inflammatory progression. On the contrary, granulocytes and monocytes tended to locate on the late branches, representing the occurrence of inflammatory infiltration. Meanwhile, clusters of macrophages and dendritic cells presented with highly distinct trajectories throughout the inflammatory response, suggesting these cells might have multiplex roles in the inflammation progression.

To investigate the roles these dual-feature cells might have in inflammation progression, analyses of enriched genes expressed specifically by each of the three clusters was undertaken using the GO database. *Mki67* ^+^ progenitor cells were characterized by enrichment of the pathways associated with “cell cycle”, “production and response to cytokines”, and “phagocytosis” (**Figure 6A**). Epithelial cells showed enrichment in the signaling pathways associated with “endocytosis”, “cell chemotaxis”, “cell differentiation”, and “response to inflammatory cytokines” (**Figure 6B**). Osteoblastic stromal cells showed significant enrichment in the signaling pathways associated with “bone development”, “endocytosis”, “cell chemotaxis” and, to a lesser extent, “negative regulation of inflammatory response” (**Figure 6C**). Hence, all of these dual-feature cells showed enrichment in signaling pathways associated with immune regulation and phagocytosis-related functions.

We ordered these dual-feature cells along a pseudotime trajectory to aid understanding of which developmental stages these cells might have in their immunoregulatory functions. This ordering resulted in a bifurcated early-to-late trajectory which represented the dynamic changes of the normal MEM after exposure to LPS (**Figure 6D**). These results showed that most of the epithelial cells and osteoblastic stromal cells were derived from the normal MEM, whereas *Mki67* ^+^ progenitor cells were scattered along the whole trajectory (**Figure 6E**).

Taken together, it is postulated that epithelial cells and osteoblastic stromal cells exist mainly in the normal MEM, which might help to maintain the homeostasis of the immune microenvironment through phagocytosis-related function and negative regulation of the inflammatory response. However, *Mki67* ^+^ progenitor cells might be activated and proliferate in response to proinflammatory stimuli and undertake their immunoregulatory functions throughout the whole process of inflammatory progression.

## Discussion

Using scRNA-seq, we comprehensively profiled the multicellular ecosystem in the normal MEM and monitored its dramatic alterations in response to proinflammatory stimuli. In this way, we portrayed the previously underestimated cell diversity and sophisticated interplay among different cell types.

Ryan et al. explored the “landscape” of the normal MEM by sequencing ~6770 normal MEM cells and identified 17 cell clusters (9), which is partially consistent with our results for the normal MEM. They showed that the genes associated with the innate immune system were expressed mainly by resident macrophages, which were inferred to be the “master regulators” of the response to infection by the MEM. However, they did not validate their hypothesis by sequencing cells in the inflamed MEM and systematically investigating the alterations from the normal MEM. Thus, we further tested their postulation by analyzing the cell–cell interactions in the inflamed MEM.

We observed that the number of interactions with macrophages was obviously higher than that with other clusters. More specifically, significant somatotrophic interactions among macrophages were observed, including CSF1–CSF1R and IL34–CSF1R. These observations support the concept of macrophage *niches* in the MEM that mediate the maintenance and proliferation of macrophages in response to external stimuli (28). Besides, significant interactions related to chemoattraction (e.g., CCL3–CCR5 and CXCL1–CXCR2) and regulation of the proinflammatory/anti-inflammatory phenotypes of neutrophils (IL1β–IL1R2) were observed among macrophages and other immune cells (mainly neutrophils) (29). Overall, these data strongly suggest that macrophages might be the driving force of innate immunity in the inflammatory response of the MEM, and are endowed with self-regeneration capacity by the formation of macrophage *niches*.

Interestingly, heterogeneous groups of dual-feature cells with phagocytic function were identified in our study. They had the phenotypes of a certain cell identity and functions related to phagocytosis or endocytosis. These dual-feature cells mainly camouflaged themselves as epithelial cells and endothelial cells, which is consistent with the definition of non-professional phagocytes described previously (39). The crosstalk disruption between professional phagocytes and non-professional phagocytes has been shown to be responsible for various diseases, and might play an important part in the inflammatory response (38), which was confirmed in the MEM in our study. Specifically, the IGF1 signaling pathway was enriched in the crosstalk among macrophages and epithelial-phagocytic dual-feature cells in the normal MEM, whereas it was clearly dampened in the inflammatory condition.

Moreover, we observed that macrophages were more likely to have a M2 phenotype in the normal MEM than in the inflamed MEM, which is consistent with the activation patterns and corresponding function of macrophages (19, 20, 41). Besides, studies have revealed that M2 macrophages can secrete IGF1 to redirect phagocytosis by non-professional phagocytes and, thus, influence inflammation progression (30). Therefore, we postulate that the crosstalk among epithelial-phagocytic dual-feature cells and M2 macrophages through the IGF1 signaling pathway might influence the inflammation progression of AOM. However, the few sampling timepoints prevented us from monitoring the dynamic changes of macrophage polarization and IGF1 signaling pathway-related cellular crosstalk along the entire process of inflammation progression.

Overall, our single-cell sequencing study revealed AOM to be characterized by rapid infiltration of innate immune cells in the MEM. We identified phagocytosis-related dual-feature cell clusters in the inflamed MEM and normal MEM, of which M2 macrophages were crucial in the network of regulatory intercellular crosstalk during inflammation progression. The exact mechanisms of these processes remain to be elucidated, but our results provide more precise understanding of AOM at the molecular level, and proffer more information for deciphering the pathogenic pathways.

## Data Availability Statement

The data presented in the study are deposited in the NCBI SRA repository, accession number PRJNA757973.

## Ethics Statement

The animal study was reviewed and approved by the Animal Ethics Committee of West China Hospital within Sichuan University (Chengdu, China).

## Author Contributions

JR, HY, and YuZ contributed to the study conception and design. Material preparation was performed by YR, DZ, KQ, YiZ, and LL. Data collection was performed by KQ, DZ, DC, MM, WP, DL, JL, YD, and WZ. And analyses were performed by YR, DZ, YS, DC, and DL. The first draft of the manuscript was written by KQ, YR, DZ, and DC, and all authors commented on previous versions of the manuscript. All authors contributed to the article and approved the submitted version.

## Funding

This work was supported by West China Hospital, Sichuan University (YZ, grant #2019HXFH003, grant #ZYJC21027); Chengdu Science and Technology Bureau (JR, grant #20GJHZ0193); Sichuan University (YZ, grant #20ZDYF3010, JR, grant #2019HXBH079, #2020SCU12049); The Science and Technology Department of Sichuan Province (YZ, grant #2020YFH0090, JR, grant#2020YFS0111); The Health Department of Sichuan Province (JR, grant #20PJ030); China Postdoctoral Science Foundation (JR, grant #2020M673250); National Natural Youth Science Foundation of China (JR, grant #82002868).

## Conflict of Interest

The authors declare that the research was conducted in the absence of any commercial or financial relationships that could be construed as a potential conflict of interest.

## Publisher’s Note

All claims expressed in this article are solely those of the authors and do not necessarily represent those of their affiliated organizations, or those of the publisher, the editors and the reviewers. Any product that may be evaluated in this article, or claim that may be made by its manufacturer, is not guaranteed or endorsed by the publisher.

## Supplementary Material

Supplementary Information 1 Clustering strategies of all single cells from both normal and inflamed MEM.

Supplementary Information 2 Clustering strategies of CD68+ single cells from both normal and inflamed MEM.

Supplementary Table 1 Summary of regents used in this study.

Supplementary Table 2 Summary of cell numbers of identified eight major cell types.

Supplementary Table 3 Summary of cell numbers of identified 20 cell clusters.

Supplementary Table 4 Summary of differentially expressed gene lists of identified 20 cell clusters.

Supplementary Table 5 Summary of cell-to-cell communications between identified 20 cell clusters.

Supplementary Table 6 Summary of differentially expressed gene lists of identified cell clusters at different resolutions.

Supplementary Table 7 Summary of cell numbers of identified 12 CD68^+^ cell clusters at the resolution of 0.6.

Supplementary Table 8 Summary of gene panels used for the calculation of cell scores.

Supplementary Figure 1
**(A)** The developmental trajectory of all cells inferred by Monocle2 across pseudotime **(B)** Kinetics plot showing relative expression of selected marker genes across developmental pseudotime.

Supplementary Figure 2 The inferred signaling networks between different cell clusters from inflamed and normal MEM, respectively. Circle sizes are proportional to the number of cells in each cell group and edge width represents the communication probability.

Supplementary Figure 3
**(A)** Clustering tree showing the relationship between clusters at different resolutions. **(B)** UMAP plot showing doublets and singlets from all CD68^+^ cells **(C)** UMAP plot of CD68+ single cells at different resolutions.

Supplementary Figure 4
**(A)** Expression of marker genes in defined clusters **(B)** Scatterplot showing the correlation between the M1 and M2 scores of single cells from the four distinct macrophage subclusters **(C)** Comparison of M1 and M2 scores of single cells between the four distinct macrophage subclusters.
