# Supplementary material for: Single-Cell Transcriptome Profiling Identifies Phagocytosis-Related Dual-Feature Cells in A Model of Acute Otitis Media in Rats
Source: Front Immunol. 2021 Oct 25;12:760954. doi: 10.3389/fimmu.2021.760954 (PMC8572853; doi:10.3389/fimmu.2021.760954)
Supplement: Supplementary Information 1 — Clustering strategies of all single cells from both normal and inflamed MEM. [file DataSheet_1.zip › Supplementary information.docx]

**Supplementary information 1. Clustering strategies of all single cells from both normal and inflamed MEM.**

Clusters C0, C2, C3 and C9 displayed high expression levels of *S100a9* and *Mmp9*, typical makers of neutrophils (Figure 1H, Table S4). Clusters C1 and C13 displayed high expression of macrophage-specific markers *Adgre1* (F4/80 antigen), *Cd86* (expressed by antigen-presenting cells) and *Csf1r* (controlling the production, differentiation, and function of macrophages), thus representing macrophages (Figure 1H, Table S4). Notably, cluster C1 specifically expressed Mrc1 (a marker gene of M2 macrophages), indicating its polarization toward M2 phenotype. Besides, clusters C4, C6 and C8 displayed high expression of *Mme* (involved in collagen chain trimerization) and *Col11a1* (encoding one of the two alpha chains of type XI collagen), thus were likely to represent fibroblasts (Figure 1H, Table S4). High expression of *Epcam* (the epithelial cell adhesion gene) was detected in clusters C5, C7, C11, C18 and C19, indicating their intimate relationships with epithelial cells (Figure 1H, Table S4). However, these clusters constituted a highly heterogeneous group with their own unique genetic characteristics. Exemplified by clusters C5 and C11, which specifically expressed certain secretory antibacterial peptides and chemoattractant for neutrophils, including CXCL3 and CXCL6, thus were defined as secretory epithelial cells (Figure 1H, Table S4). Similarly, cluster 19 specifically expressed many genes characteristic of ciliated cells(Hoh et al., 2012), including *Hydin* (axonemal central pair apparatus protein) and the dynein regulator gene *Dynlrb2* (dynein light chain roadblock 2) (Figure 1H, Table S4). Besides, cluster 18 also specifically expressed *Top2a* (involved in cell cycle) and *Mki67* (marker gene of cellular proliferation), thus representing a subpopulation of epithelial cells with high proliferating capacity (Figure 1H, Table S4). In addition, cluster 7 specifically expressed *Tp63*, which was previously proved to be a cell marker of basal cell in prostate and submandibular gland(Signoretti et al., 2000; Lu and Gao, 2007), thus was defined as basal epithelial cells. Cluster 10 displayed high expression levels of T cell receptor gene *Cd3e* (Figure 1H, Table S4), while cluster 17 displayed high expression levels of many genes related to B-cell function, including *Jchain* (joining chain of multimeric IgA and IgM) and *Ighm* (an antigen recognition molecule of B cells) (Figure 1H, Table S4). Cluster 12 displayed high expression levels of many genes related to antigen presenting function, including *Cd83*, *RT1-Bb, RT1-Db1,* *RT1-Da* and *RT1-Ba*. However, macrophage specific marker genes such as *Adgre* and *Mrc1* were not detected in cluster 12, thus we postulated it to be dendritic cells (DCs) (Figure 1H, Table S4). Both clusters C14, C15 and C16 all displayed high expression of many genes related to endothelial cell function but with different patterns, thus representing 3 heterogeneous subpopulations of endothelial cells. Among them, cluster 14 specifically expressed *Flt4* (involved in lymph-angiogenesis and maintenance of the lymphatic endothelium) and *Lyve1* (lymphatic vessel endothelial hyaluronan receptor 1), thus was defined as lymphatic endothelial cells (Figure 1H, Table S4). Similarly, Cluster 15 specifically expressed *Mcam* (involved in cohesion of the endothelial monolayer at intercellular junctions in vascular tissue) and *Rgs5* (involved in the regulation of blood pressure), thus was defined as pericytes (Figure 1H, Table S4). Cluster 16 specifically expressed *Flt1* (a typical marker gene of vascular endothelial cells), thus was defined as vascular endothelial cells (Figure 1H, Table S4).

**Supplementary information 2. Clustering strategies of CD68^+^ single cells from both normal and inflamed MEM.**

Clusters C0, C1, C5 and C10 displayed high expression of macrophage-specific markers *Adgre1* (F4/80 antigen), thus representing macrophages (Figure 3D, Table S14-4). Among them, cluster C5 displayed obviously lower expression of both *Ifngr1* and *Il4r*, which are necessary for the activation of macrophages, thus cluster C5 was defined as senescent macrophages (Figure 3D, Table S14-4). Besides, both of cluster C0 and C1 displayed high expression of *Mrc1* (a typical marker gene of M2 macrophages), indicating their polarization toward M2 phenotype (Figure 3D, Table S14-4). Notably, many of cells in cluster C0 specifically expressed *C1qc* (c-chain polypeptide of serum complement subcomponent C1q), which was proved to mediate similar biological functions to TNFα in the manners of autocrine or paracrine.(Ghebrehiwet et al., 2017) Similarly, cluster C1 specifically expressed *Arg1* (a critical regulator of innate and adaptive immune responses), further clarifying their contributions to the resolution of inflammation and wound repair.(Burrack and Morrison, 2014) (Figure 3D, Table S14-4). Besides, moderate expression of both marker gene of M2 macrophages and M1 macrophages was observed in cluster C10, thus C10 was defined as intermediate macrophages (Figure 3D, Table S14-4). Additionally, clusters C4, C6 and C11 preferentially displayed high expression of mature dendritic cell markers *Cd83* and *Itgax*, and also genes involved in antigen presenting, including RT1-Bb, RT1-DOa and RT1-Ba, consistent with dendritic cell identity (Figure 3D, Table S14-4). Interestingly, many of cells in cluster C4 specifically expressed *Lyve1* (Lymphatic Vessel Endothelial Hyaluronan Receptor 1), which was previously thought to be exclusively expressed by lymphatic endothelial cells and identified as a lymphatic docking receptor, indicating that certain dendritic cells might also play a mysterious part in leukocytes trafficking (Figure 3D, Table S14-4).(Jackson, 2019) Similarly, cluster C6 specifically expressed *Fcgr3a* (Fc Fragment Of IgG Receptor IIIa), highly suggesting that these cells might be involved in mediating certain antibody-dependent responses including cellular cytotoxicity (Figure 3D, Table S14-4). In addition, cluster C11 specifically expressed *Clec9a*, consistent with the identity of a newly identified subpopulation named cross-presenting dendritic cells (Figure 3D, Table S14-4).(Villani et al., 2017) The remained non-dendritic cell and non-macrophage monocytes might represent a mix of different subpopulations, which constitutes cluster C2 (Figure 3D, Table S14-4). What’s more, cluster C3 preferentially displayed high expression of genes related to the function of granulocytes, including *Csf3r* (controlling the production, differentiation, and function of granulocytes), *Cxcr2* (mediating neutrophil migration) and *Srgn* (a mediator of granule-mediated apoptosis), which was consistent with the identity of granulocytes (Figure 3D, Table S14-4). Notably, cluster C7 displayed high expression of cell cycle related genes including *Ccna2*, *Ccnb1* and *Cdk1*, as well as stem-like genes including *Mki67* (marker gene of proliferation) and *Aspm* (involved in mitotic spindle regulation and coordination of mitotic processes), thus was defined as Mki67^+^ progenitor cells (Figure 3D, Table S14-4).(Gao et al., 2018) Besides, cluster C8 specifically expressed epithelial cell adhesion gene *Epcam* and the cytokeratin genes *Krt18*, highly indicating these cells were epithelial cells (Figure 3D, Table S14-4). And high expression of genes related to bone development and maintenance, including *Bmp5* and *Cdh11*, as well as genes related to matrix homeostasis including Col5a2, Col12a1 and Mmp2, were observed in cluster C9, thus were defined as osteoblastic stromal cells (Figure 3D, Table S14-4).

**Reference**

Burrack, K.S., and Morrison, T.E. (2014). The role of myeloid cell activation and arginine metabolism in the pathogenesis of virus-induced diseases. *Front Immunol* 5**,** 428. doi: 10.3389/fimmu.2014.00428.

Gao, S., Yan, L., Wang, R., Li, J., Yong, J., Zhou, X., et al. (2018). Tracing the temporal-spatial transcriptome landscapes of the human fetal digestive tract using single-cell RNA-sequencing. *Nat Cell Biol* 20(6)**,** 721-734. doi: 10.1038/s41556-018-0105-4.

Ghebrehiwet, B., Hosszu, K.H., and Peerschke, E.I. (2017). C1q as an autocrine and paracrine regulator of cellular functions. *Mol Immunol* 84**,** 26-33. doi: 10.1016/j.molimm.2016.11.003.

Hoh, R.A., Stowe, T.R., Turk, E., and Stearns, T. (2012). Transcriptional program of ciliated epithelial cells reveals new cilium and centrosome components and links to human disease. *PLoS One* 7(12)**,** e52166. doi: 10.1371/journal.pone.0052166.

Jackson, D.G. (2019). Hyaluronan in the lymphatics: The key role of the hyaluronan receptor LYVE-1 in leucocyte trafficking. *Matrix Biol* 78-79**,** 219-235. doi: 10.1016/j.matbio.2018.02.001.

Lu, Y.H., and Gao, Y. (2007). [Expression of calponin and P63 in human submandibular glands]. *Hua Xi Kou Qiang Yi Xue Za Zhi* 25(1)**,** 19-21.

Signoretti, S., Waltregny, D., Dilks, J., Isaac, B., Lin, D., Garraway, L., et al. (2000). p63 is a prostate basal cell marker and is required for prostate development. *Am J Pathol* 157(6)**,** 1769-1775. doi: 10.1016/s0002-9440(10)64814-6.

Villani, A.C., Satija, R., Reynolds, G., Sarkizova, S., Shekhar, K., Fletcher, J., et al. (2017). Single-cell RNA-seq reveals new types of human blood dendritic cells, monocytes, and progenitors. *Science* 356(6335). doi: 10.1126/science.aah4573.
