# Supplementary material for: Single-Cell Transcriptome Profiling Identifies Phagocytosis-Related Dual-Feature Cells in A Model of Acute Otitis Media in Rats
Source: Front Immunol. 2021 Oct 25;12:760954. doi: 10.3389/fimmu.2021.760954 (PMC8572853; doi:10.3389/fimmu.2021.760954)
Supplement: Supplementary Information 1 — Clustering strategies of all single cells from both normal and inflamed MEM. [file DataSheet_1.zip › Supplementary Tables/Table S1_reagent_1.docx]

Table1. Key Resources

| **Reagent or Resources** | **Source** | **Catalog No.** | **Size** |
| --- | --- | --- | --- |
| Bioactive regents |  |  |  |
| Collagenase Type I | Sigma-Aldrich | SCR103 | 250 mg |
| Dispase®II(neutral protease, grade II) | Sigma-Aldrich | 4942078001 | 5 x 1 g |
| Accumax Cell Dissociation Solution | Innovative Cell Technologies | AM105 | 100 ml |
| RPMI-1640 Medium | Sigma-Aldrich | R8758 | 500 ml |
| DPBS Buffer Solution | Sigma-Aldrich | D8662 | 500 ml |
| MACS Tissue Storage Solution | MiltenyiBiotec (Germany) | 130-100-008 | 100 ml |
| 4% PFA Fix Solution | Beyotime Biotechnology | P0099 | 100 ml |
| EDTA Decalcified Solution | Biosharp | BL616A | 100 ml |
| Calcein AM (cell-permeant dye) | Thermo Fisher Scientific | C1430 | 1 mg |
| DRAQ7™ 0.3 mM | BD Biosciences | 564904 | 1 ml |
| BD Rhapsody™ Targeted Reagent Kit | BD Biosciences | 633730 | 4 pack |
| BD Rhapsody™ Targeted Training Kit | BD Biosciences | 633740 | 4 pack |
| BD Rhapsody™ Custom Panel Design | BD Biosciences | 633743 | 4 pack |
| Ethyl alcohol, Pure (200 proof, molecular biology grade) | Sigma-Aldrich | E7023 | 500 ml |
| Stain Buffer (FBS) | BD Biosciences | 554656 | 500 ml |
| Agencourt® AMPure® XP magnetic beads |  |  |  |
| Antibodies |  |  |  |
| Manual Opal 7-Color IHC Kit | Akoya Biosciences | NEL811001KT | 50 slides |
| Recombinant Anti-EpCAM antibody [EPR20532-225] | Abcam | ab223582 | 10 µl Trial Size |
| CDK4 (D9G3E) Rabbit mAb | Cell Signaling Technology | 12790 | 20 µl |
| Recombinant Anti-CD68 antibody [KP1] | Abcam | ab955 | 10 µl |
| Recombinant Anti-Collagen I antibody [EPR24331-53] | Abcam | ab270993 | 10 µl |
| Facilities |  |  |  |
| BD Rhapsody™ Scanner | BD Biosciences | 633701 | N/A |
| BD Rhapsody Express instrument | BD Biosciences | 633702 | N/A |
| 6-Tube Magnetic Separation Rack for 1.5 mL tubes | New England Biolabs | S1506S | N/A |
| Large magnetic separation stand | V&P Scientific | VP 772FB-1 | N/A |
| Clear acrylic cylinder adapter for 15 mL tube magnet | V&P Scientific | VP 772FB-1A | N/A |
| Qubit™ 3.0 Fluorometer | Thermo Fisher Scientific | Q33216 | N/A |
| Software |  |  |  |
| BD Genomics Rhapsody Analysis Pipeline CWL | https://bitbucket.org/CRSwDev/cwl/src/master/ | v1.9.1 | N/A |
| Seurat | https://satijalab.org/seurat/ | v4.0.0 | N/A |
|  |  |  |  |
