# Supplementary figures and images for: Single-Cell Transcriptome Profiling Identifies Phagocytosis-Related Dual-Feature Cells in A Model of Acute Otitis Media in Rats
Source: Front Immunol. 2021 Oct 25;12:760954. doi: 10.3389/fimmu.2021.760954 (PMC8572853; doi:10.3389/fimmu.2021.760954)

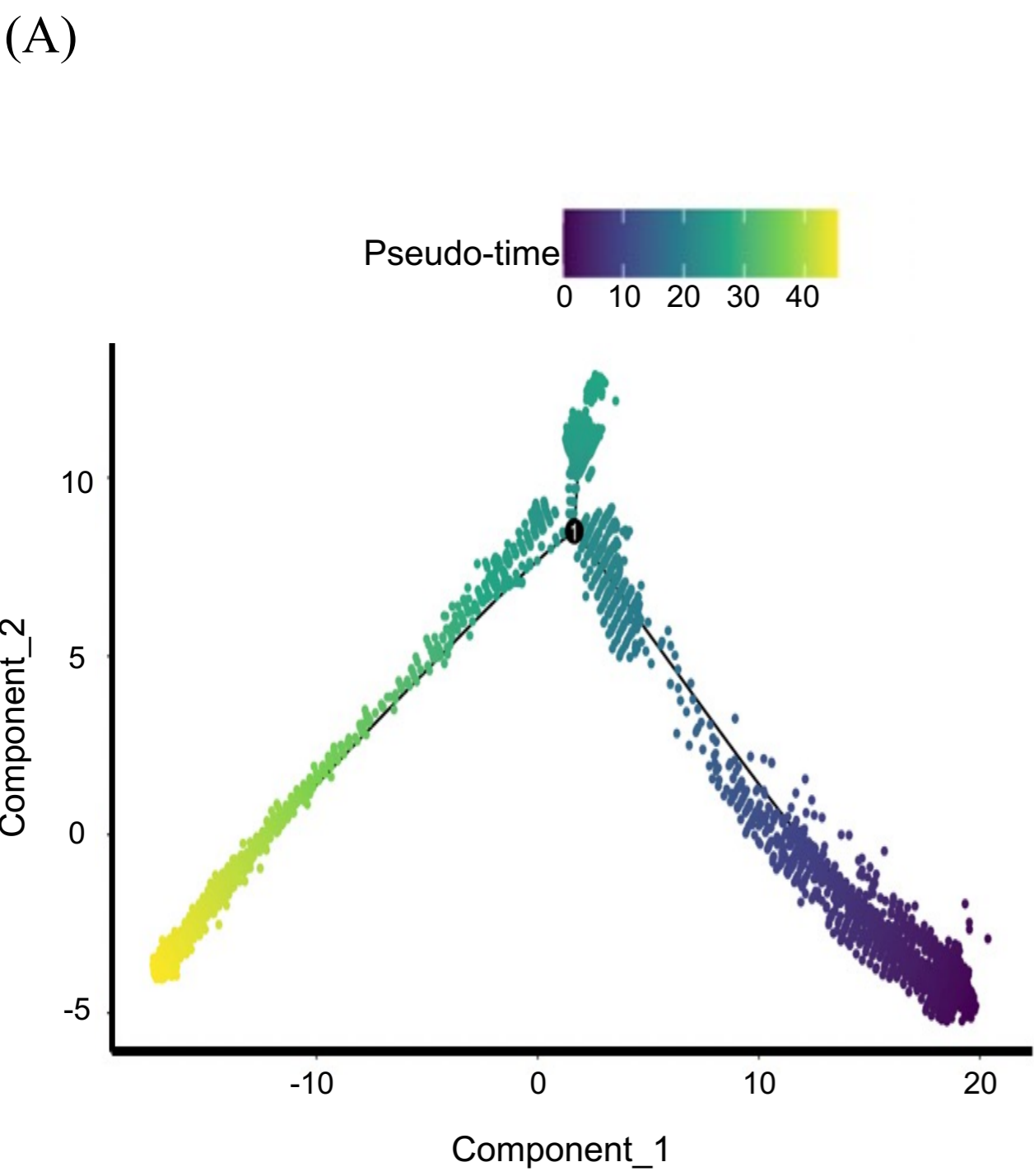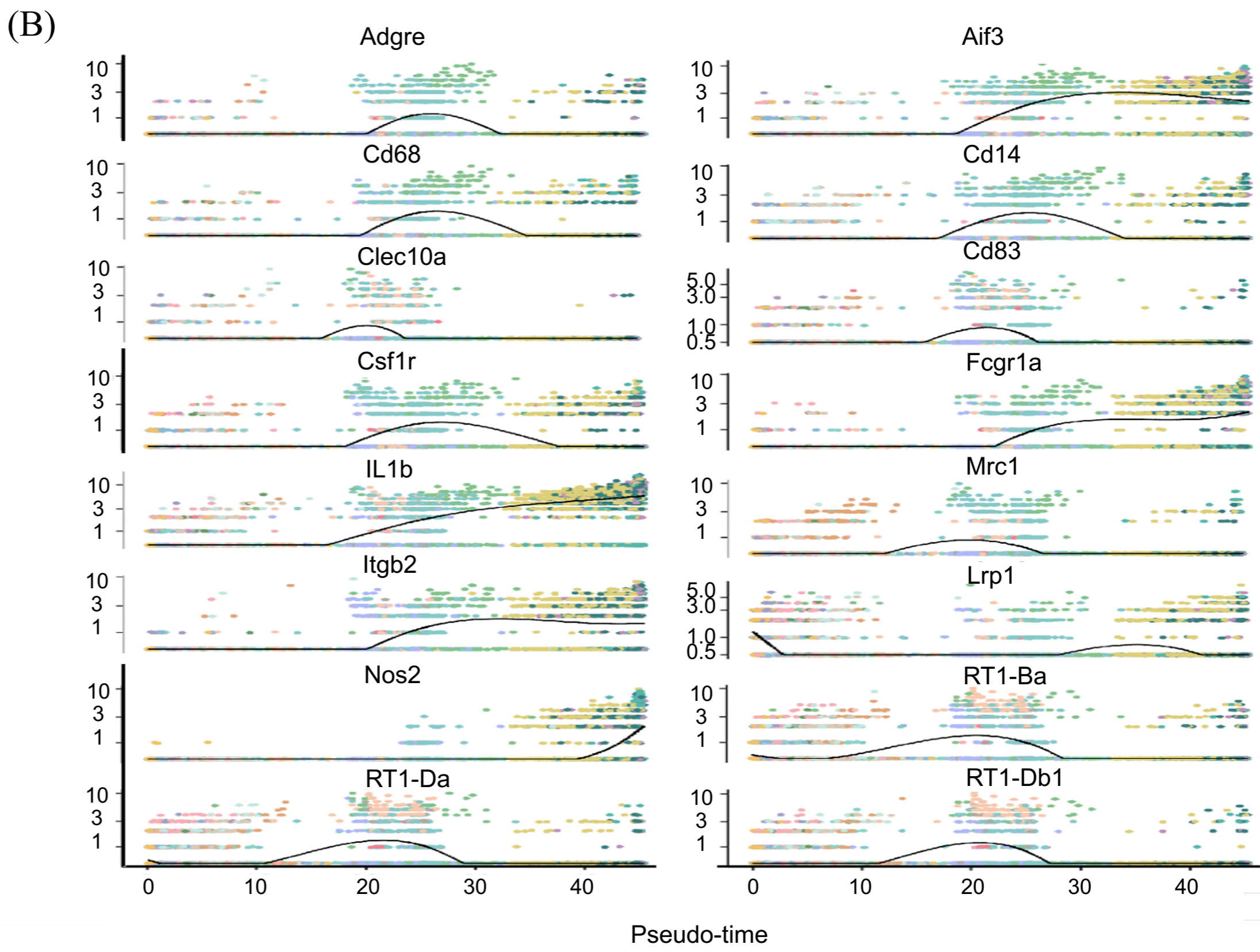

Supplement: Supplementary Information 1 — Clustering strategies of all single cells from both normal and inflamed MEM. [file DataSheet_1.zip › Supplementary Figure S1-S4/Figure S1.pdf]

Case

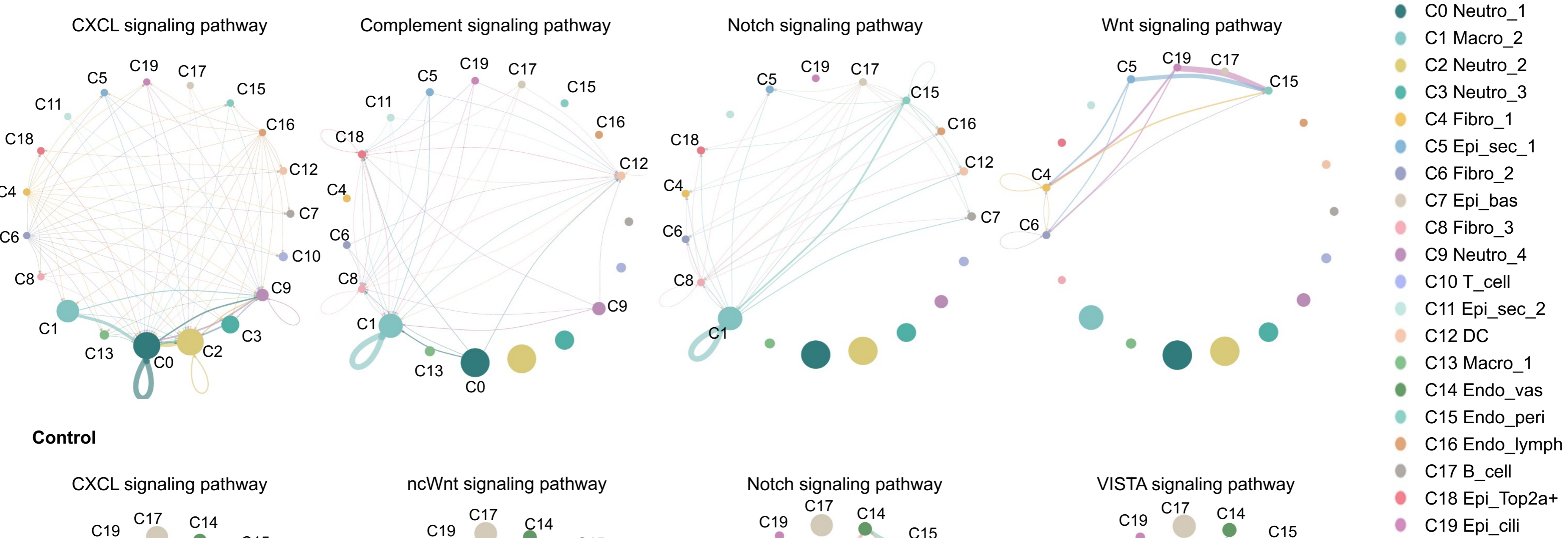

Control

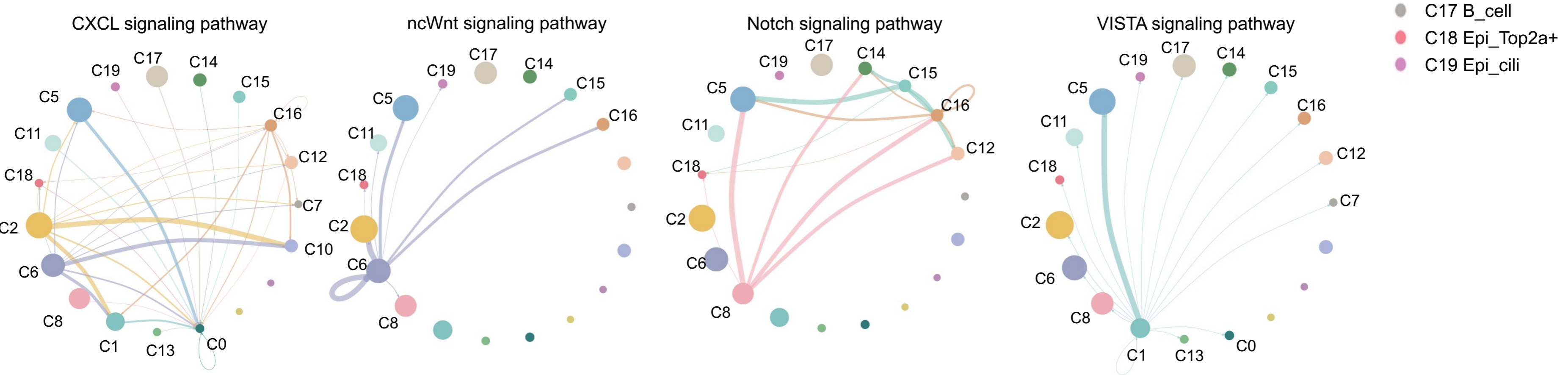

Case

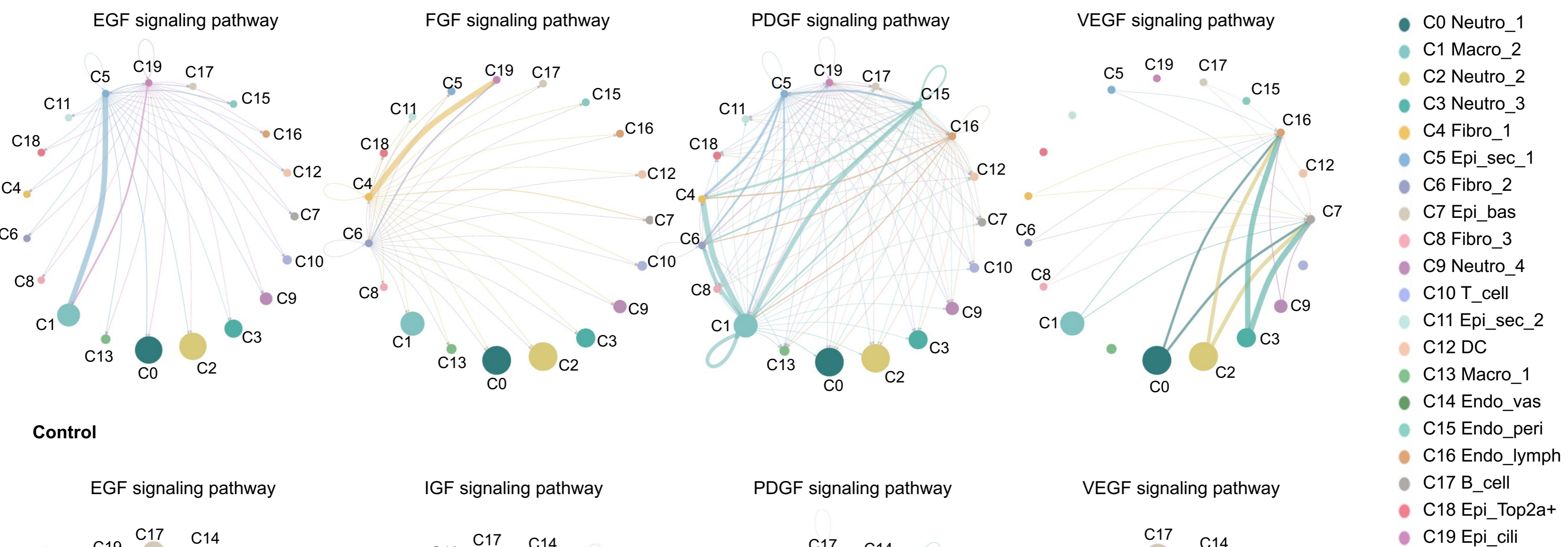

Control

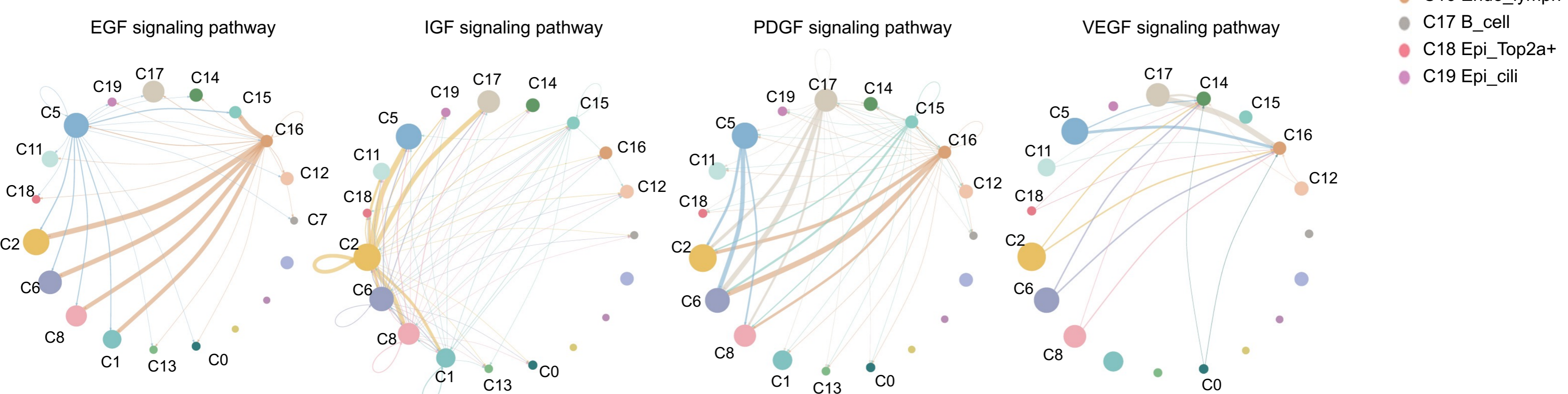

Supplement: Supplementary Information 1 — Clustering strategies of all single cells from both normal and inflamed MEM. [file DataSheet_1.zip › Supplementary Figure S1-S4/Figure S2.pdf]

(A)

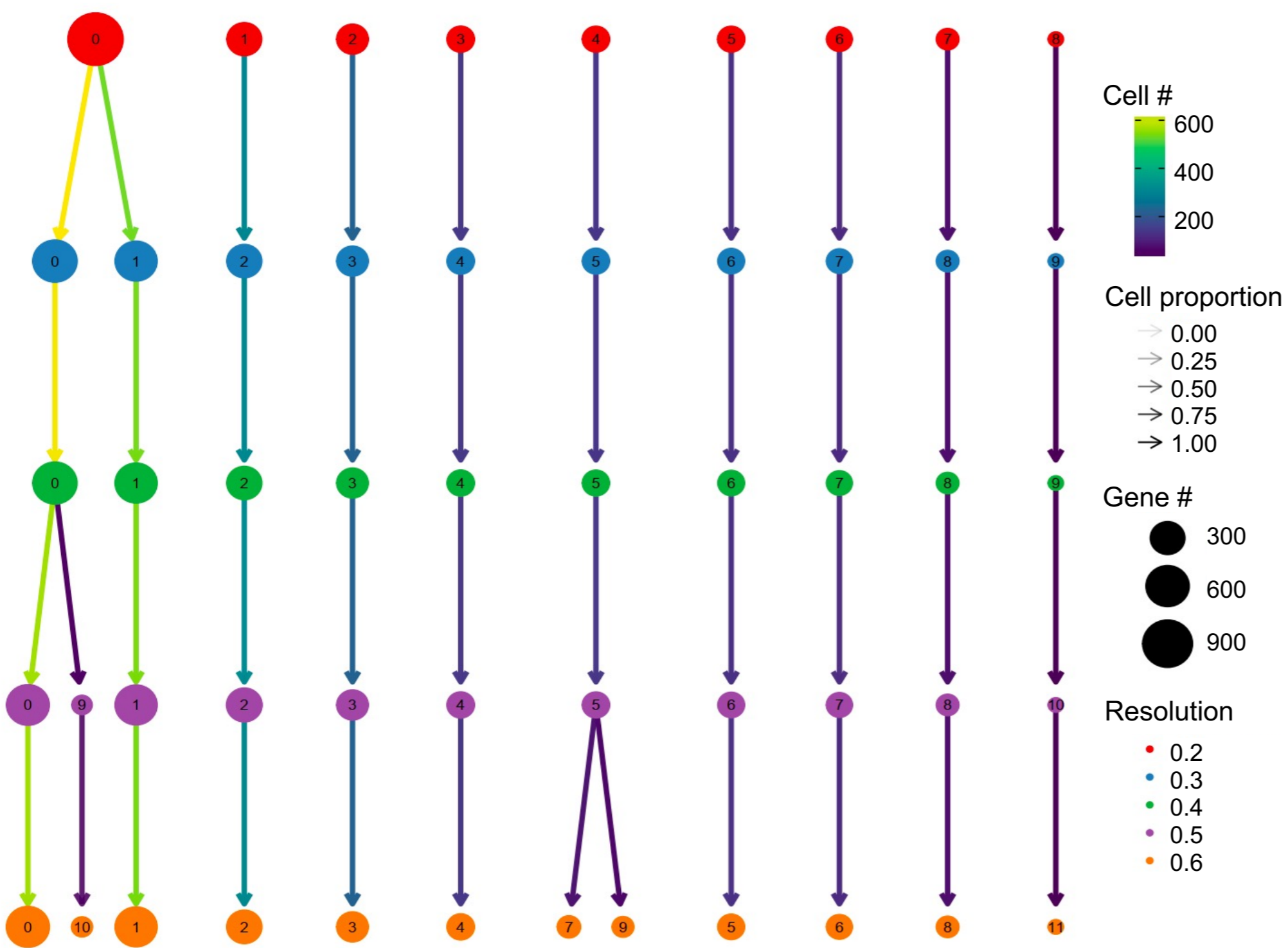

(B)

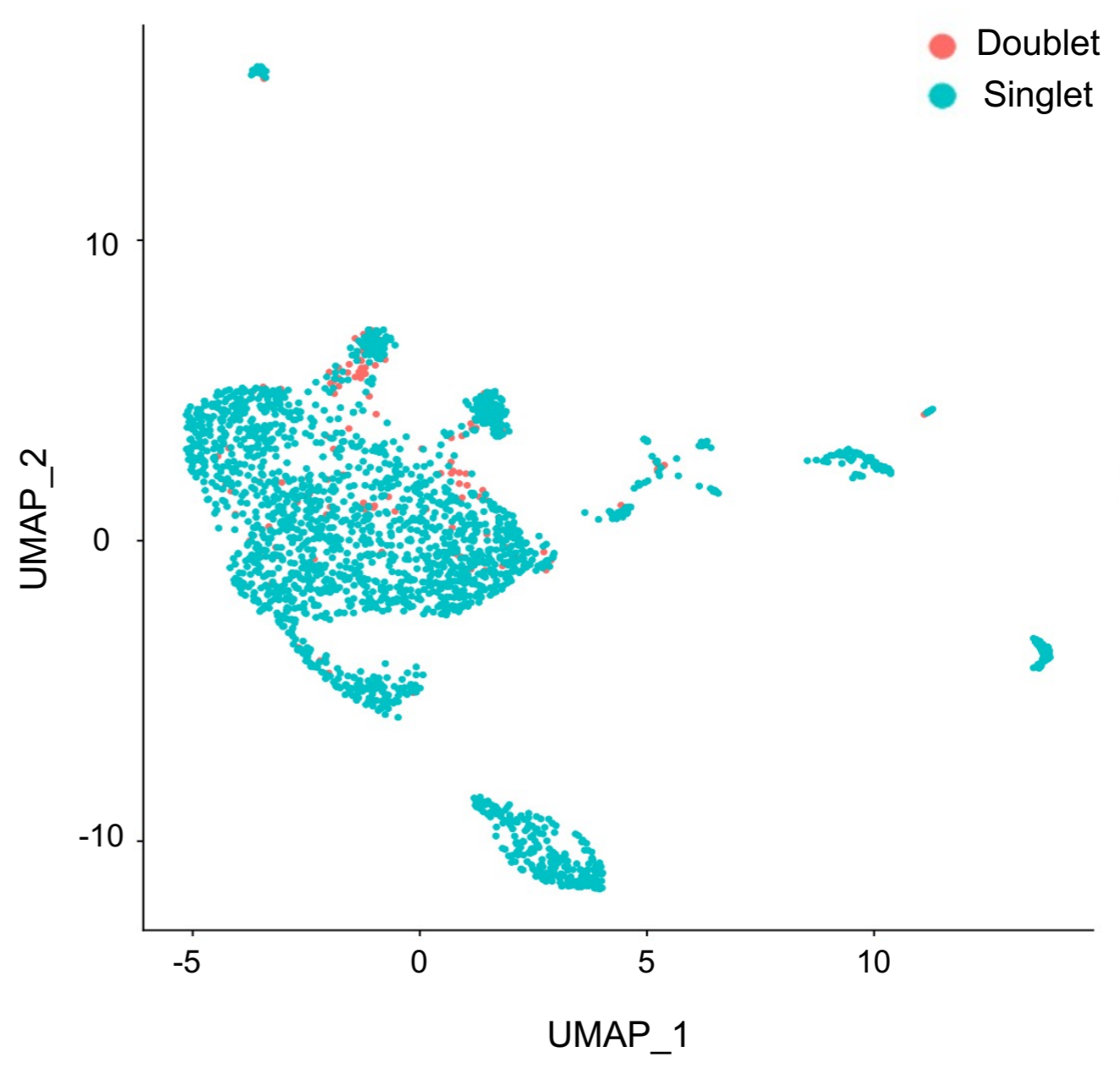

(C)

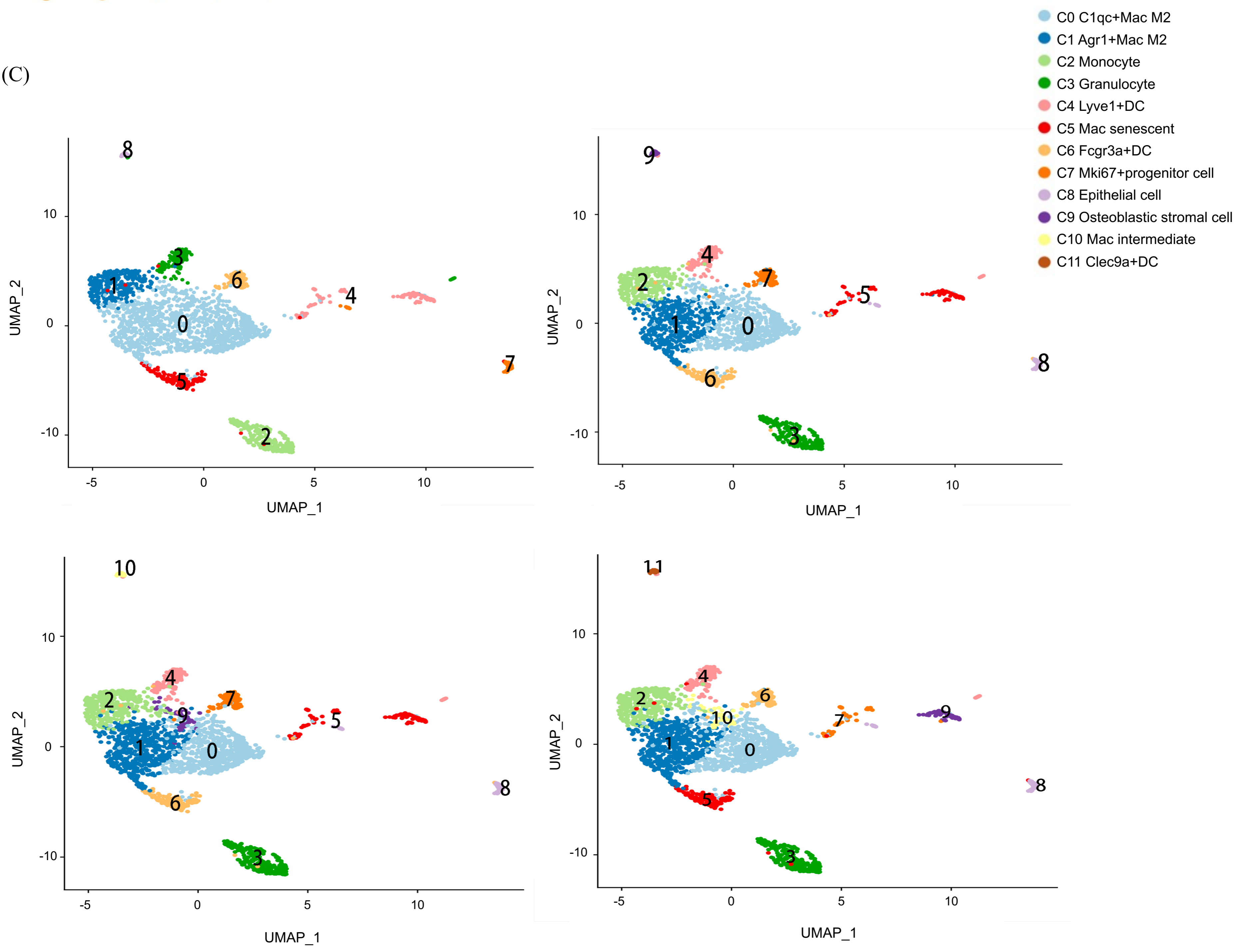

Supplement: Supplementary Information 1 — Clustering strategies of all single cells from both normal and inflamed MEM. [file DataSheet_1.zip › Supplementary Figure S1-S4/Figure S3.pdf]

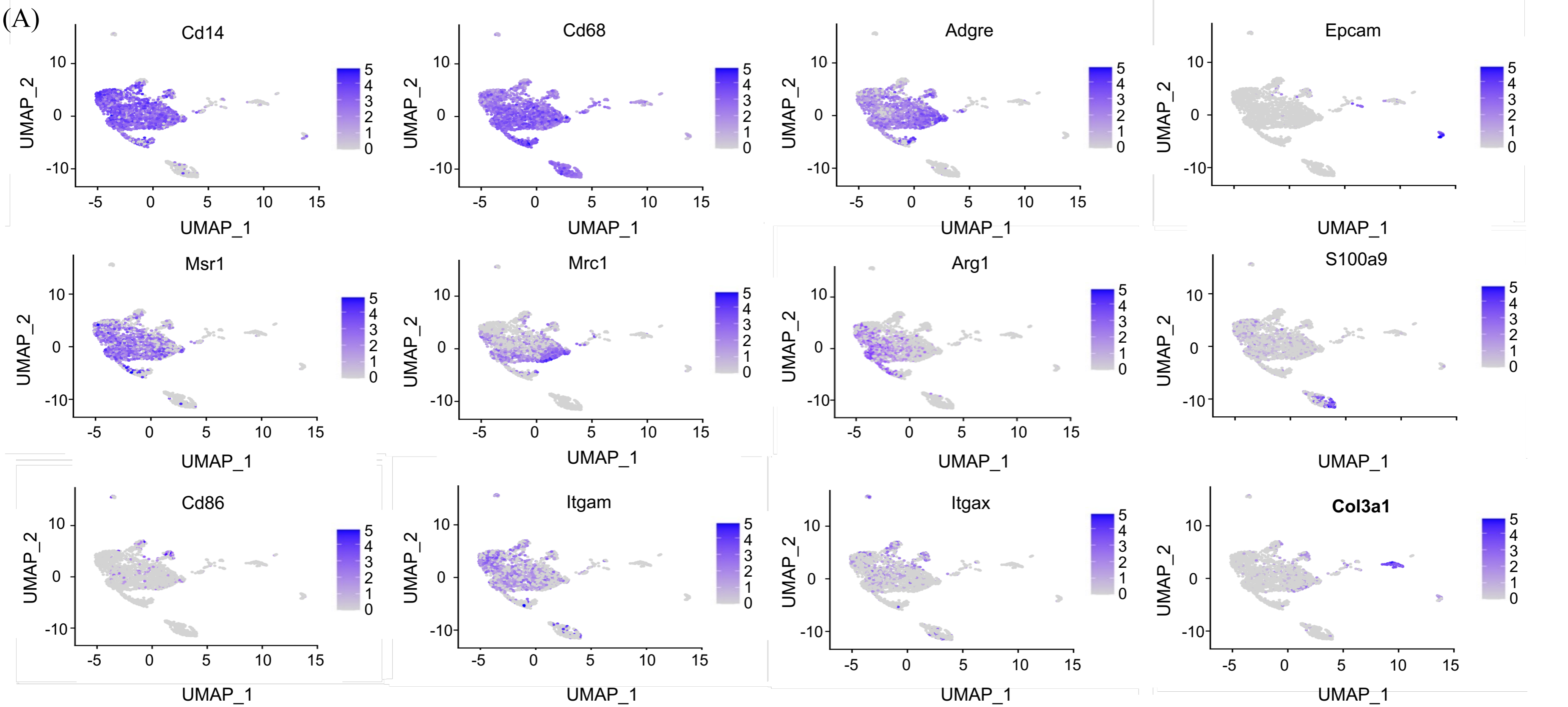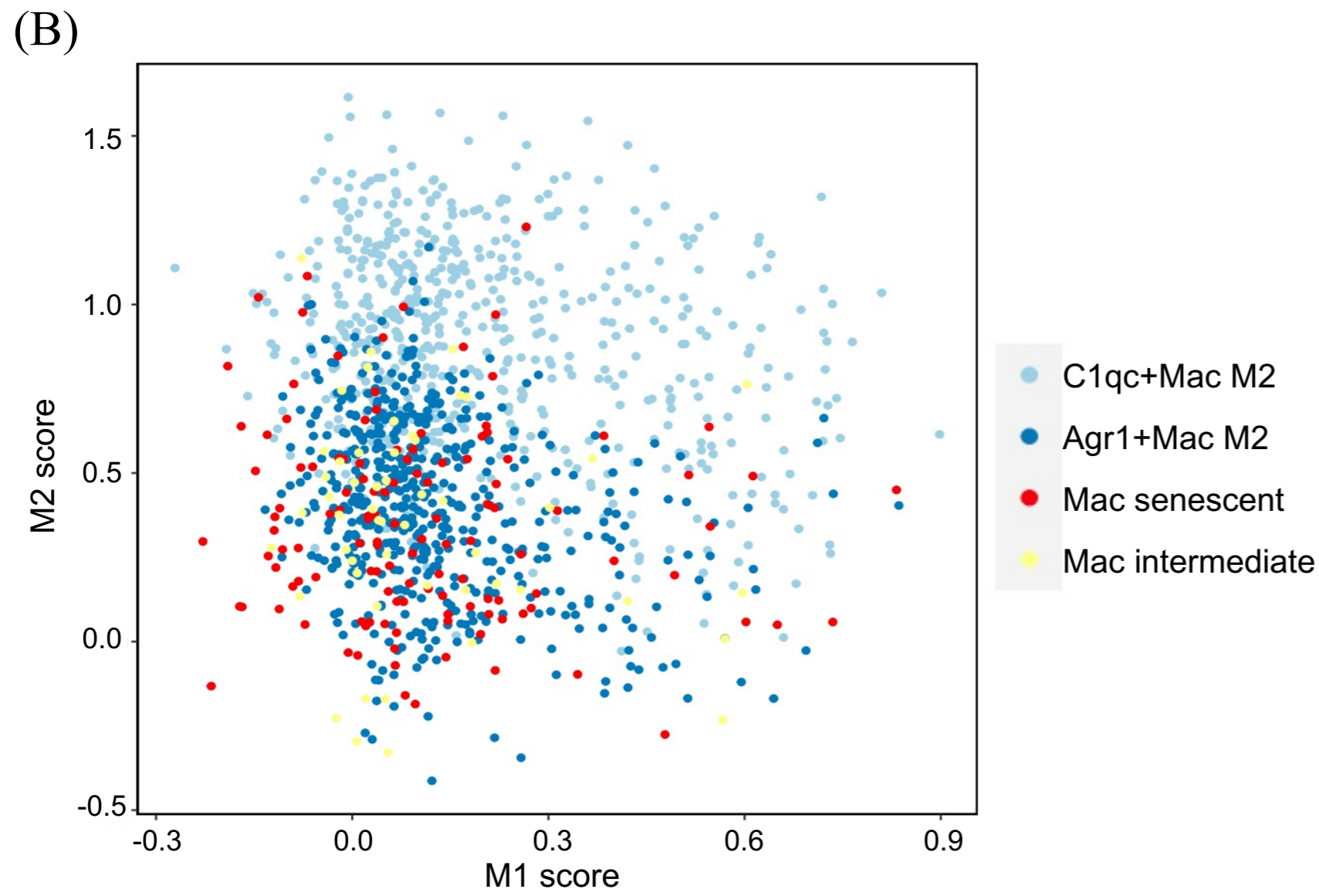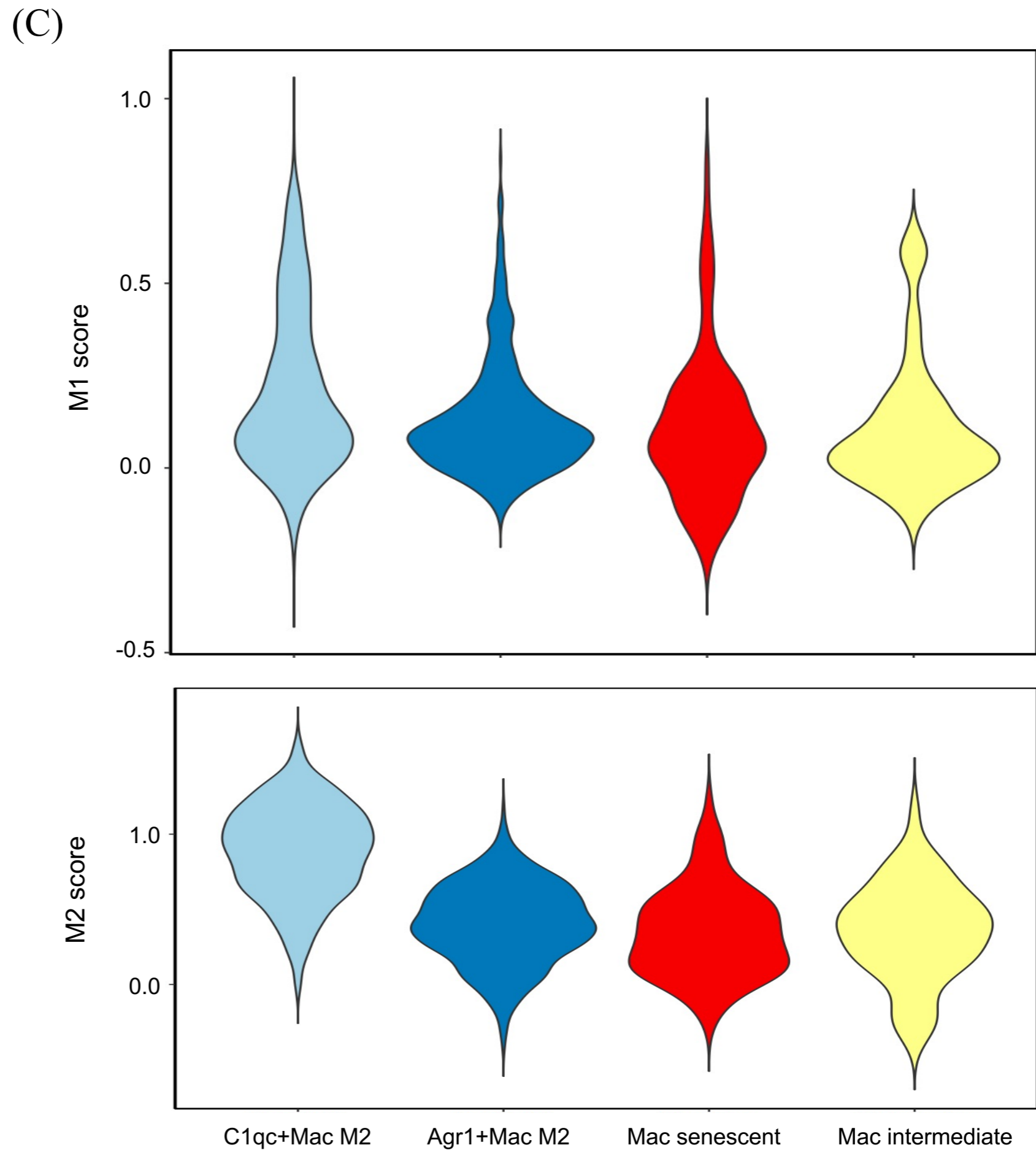

Supplement: Supplementary Information 1 — Clustering strategies of all single cells from both normal and inflamed MEM. [file DataSheet_1.zip › Supplementary Figure S1-S4/Figure S4.pdf]
